# An update on the molecular mechanisms of ZFAS1 as a prognostic, diagnostic, or therapeutic biomarker in cancers

**DOI:** 10.1007/s12672-024-01078-x

**Published:** 2024-06-10

**Authors:** Mahdieh Mehrab Mohseni, Hedyeh Zamani, Mina Momeni, Zeinab Shirvani-Farsani

**Affiliations:** Department of Cell and Molecular Biology, Faculty of Life Sciences and Biotechnology, Shahid Beheshti University, Tehran, IR Iran

**Keywords:** ZFAS1, Long noncoding RNA, Chemoresistance, Cancer, Biomarker, miRNA

## Abstract

Zinc finger antisense 1 (ZFAS1), a newly discovered long noncoding RNA, is expressed in various tissues and organs and has been introduced an oncogenic gene in human malignancies. In various cancers, ZFAS1 regulates apoptosis, cell proliferation, the cell cycle, migration, translation, rRNA processing, and spliceosomal snRNP assembly; targets signaling cascades; and interacts with transcription factors via binding to key proteins and miRNAs, with conflicting findings on its effect on these processes. ZFAS1 is elevated in different types of cancer, like colorectal, colon, osteosarcoma, and gastric cancer. Considering the ZFAS1 expression pattern, it also has the potential to be a diagnostic or prognostic marker in various cancers. The current review discusses the mode of action of ZFAS1 in various human cancers and its regulation function related to chemoresistance comprehensively, as well as the potential role of ZFAS1 as an effective and noninvasive cancer-specific biomarker in tumor diagnosis, prognosis, and treatment. We expected that the current review could fill the current scientific gaps in the ZFAS1-related cancer causative mechanisms and improve available biomarkers.

## Background

Long noncoding RNAs (lncRNA) are mysterious RNAs with more than 200 nucleotides that, although they do not encode any proteins, are functional. In comparison to coding genes, lncRNAs contain similar promoter regions and splicing sites but a greater cellular and tissue-specific distribution. Based on studies, lncRNAs adjust vital biological processes, including human growth and development, imprinting, epigenetic regulation, and alternative splicing [1].

A lot of patients all over the world are suffering from various diseases or dying from abnormalities as a result of a lack of knowledge of lncRNA function. Abnormal lncRNA expression has been detected in a variety of disorders, including, glomerular and tubulointerstitial kidney disease [1], the pathological process of CIRI [2], and especially cancer [3]. A lot of studies find the key lncRNA roles in the development of cancer via the adjustment of their downstream elements [4]. For example, lncRNAs may act as oncogenes or tumor suppressor genes in cancer [5]. They also work as diagnostic, prognostic, or therapeutic markers in human cancers [6, 7]. As a result, lncRNAs have been identified as important factors in cancer research, and the involvement of lncRNAs in the emergence of chemoresistance in cancer cells has received a lot of attention. lncRNAs target specific downstream genes linked with chemosensitivity via regulating gene transcription, splicing, and other epigenetic processes [8].

Zinc finger antisense 1 (ZFAS1), a newly discovered lncRNA, is the antisense strand of the 5' end of the protein-coding gene zinc finger NFX1-type containing 1 (ZNFX1) and the carrier of three C/D-box snoRNAs, SNORD12C, SNORD12B, and SNORD12. It is situated on chromosome 20q13 and is stably expressed in many tissues and organs [9]. In both the cytoplasm and the nucleus, ZFAS1 is expressed in at least five different isoforms. Exons 2 and 5 are shared by all isoforms and vary in size from 516 to 1006 bases [10].

**Fig. 1:**
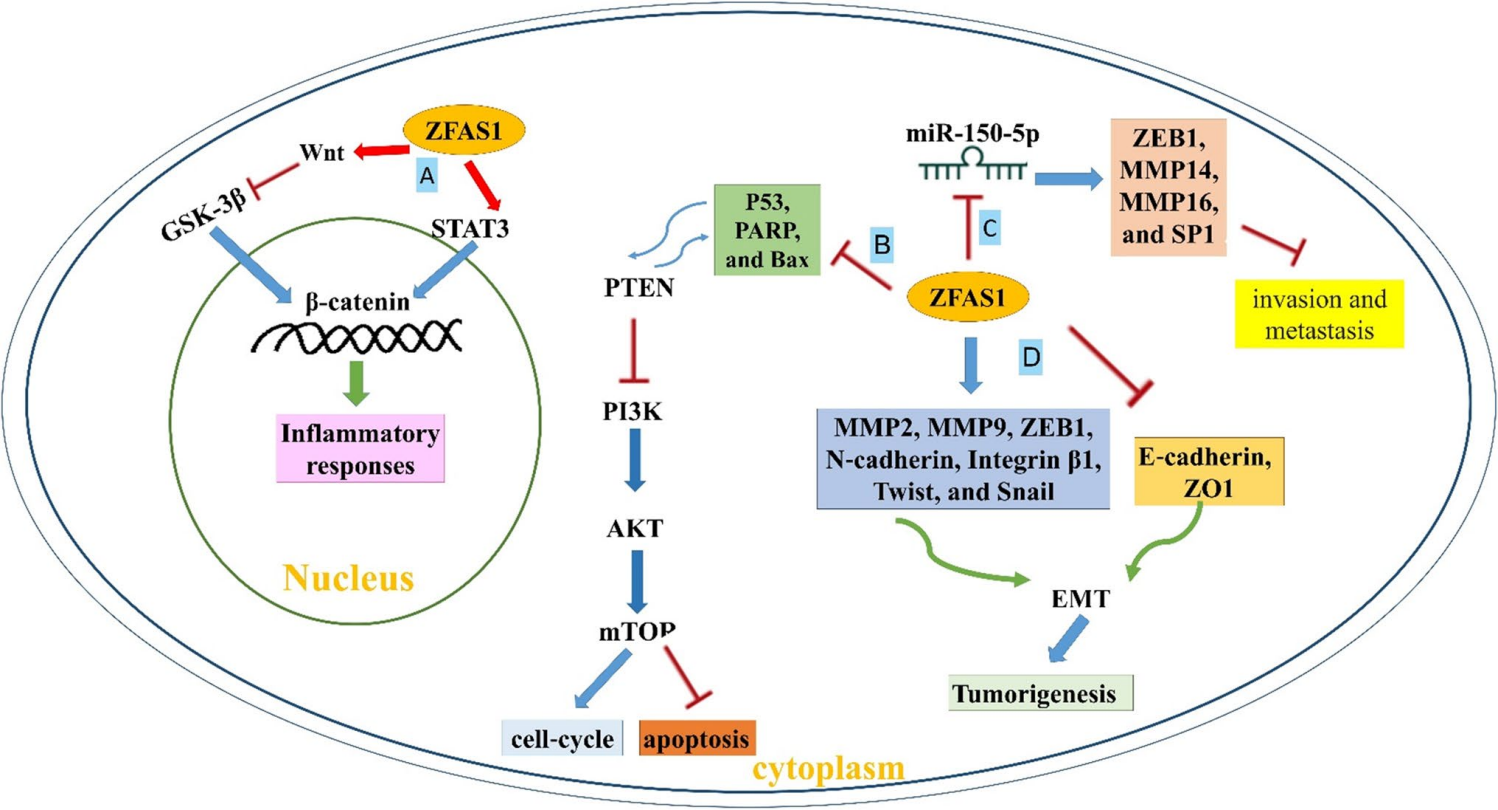
The molecular mechanisms of ZFAS1 in different human cancers. **A** ZFAS1 blocked GSK-3β via Wnt, which led to β-catenin upregulation. ZFAS1 also upregulated β-catenin via STAT3. **B** ZFAS1 increased the cell cycle and blocked apoptosis via p53, PARP, and Bax downregulation. ZFAS1 also downregulated PTEN, which blocked PI3K/AKT/mTOR, leading to cell proliferation, invasion, and migration. p53 and PTEN showed a bidirectional positive association. **C** ZFAS1 interacted with miR‐150 as a ceRNA to over-express ZEB1, MMP14, MMP16, and SP1 to block invasion and metastasis. **D** ZFAS1 upregulated the mesenchymal markers; MMP2, MMP9, N‐cadherin, Integrin β1, ZEB1, Twist, and Snail, while downregulating the epithelial markers such as E‐cadherin and ZO1, which led to EMT

ZFAS1 was initially discovered as a tumor suppressor, but the majority of studies have found it to be an oncogenic gene in human malignancies by modifying EMT (epithelial-mesenchymal transition) via targeting signaling cascades such as MAPK/ERK, PI3K/AKT, and Wnt/-catenin. ZFAS1 can regulate proliferation and migration in glioma, breast cancer, colon cancer, gastric cancer, and endometrial carcinoma via targeting E-cadherin, vimentin, MMP-2, B Lymphoma Mo-MLV Insertion Region 1 Homolog (BMI1), P21cip1, twist, snail 1/2, slug, Zeb 1/2, cyclin D1, and c-myc in various human cancer cells [11]. Figure 1 represents the molecular mechanisms of ZFAS1 in different human cancers. Several transcription factors, such as STAT3, KLF1, DDX4, ZNF274, and DDX5, are also candidates to interact with ZFAS1. In particular, ZFAS1 represents an extraordinarily long half-life (> 16 h) in mammary tissues and regulates the development of epithelial cells [8]. Table 1 shows the expression pattern of ZFAS1 and its roles in various cancers.

**Table 1:** The expression pattern of *ZFAS1* and its roles in various cancers

Cancer type	Sample type		Expression pattern	Gene interplay (direct or indirect)	Signaling pathways	Natural function	Functional role of the lncRNA	References
	Patients	Cell line						
Colorectal cancer	30 CRC and adjacent tissues, peripheral blood samples from 218 patients with CRC and 238 healthy people	SW480, RKO, HT-29, HCT116	Upregulated	PABP2, SREBP1, SCD1, and FASN	–	CRC lipid accumulation and tumorigenesis	Promoting	[12]
Colorectal cancer	157 patients’ tissues and matched tumor-adjacent control tissues	LOVO, CACO2, HT29, SW48, HCT116, SW620, and SW480	Upregulated	DDX21, POLR1b	ZFAS1/DDX21/POLR1B signaling regulation axis	Cell proliferation, invasion, migration, and apoptosis suppression	Promoting	[13]
Colorectal cancer	159 paired CRC tissues and adjacent noncancerous tissues	–	Upregulated	–	–	ZFAS1 promoted cell migration and invasion	Promoting	[14]
Colorectal cancer	119 tumor and paired non-tumor colorectal tissues	HT-29, DLD-1 (p53241F), Colo-206, CaCO-2, SW-837, and SW-620	Upregulated	cyclin B1, p53 levels, and PARP	CDK1/cyclin B1 complex	Promotes cell cycle and inhibits apoptosis	Promoting	[15]
Colorectal cancer	157 tumor and paired non-tumor colorectal tissues	HCT116, SW480, SW620, and HT29	Upregulated	NOP58, SNORD12C/78	–	ZFAS1 induced cell proliferation and migration and inhibited apoptosis	Promoting	[16]
Colonic cancer	73 paired colonic cancer tissues	–	Upregulated	ZFAS1 and ZEB1	–	ZFAS1 induced proliferation, invasion, and inhibited apoptosis	Promoting	[17]
Hepatocellular carcinoma	20 healthy liver samples, 41 PVTT, and paired primary HCC tissues as well as GSE58043 and GSE55191 databases	Huh7, HepG2, Hep3B, SMMC7721, LM3 hepatoma cell lines, and LO2, QSG7701 immortalized hepatic cells	Upregulated	ZEB1, MMP14, and MMP16	–	Cell migration, invasion, tumor recurrence, and metastasis in HCC	Promoting	[18]
Hepatoblastoma	70 paired HB tissues and adjacent non-tumor samples	The HB cell lines (HepG2 and HuH-6), normal liver cell lines (Chang liver and L02) and embryonic kidney cell lines (HEK293)	Upregulated	RALY	HGF/c-Met pathway	HB cell proliferation, migration, and invasion	Promoting	[19]
Gastric cancer	Gastric cancer tissues	MKN-28	Upregulated	EMT, Kruppel Like Factor 2 (KLF2), zeste homolog 2 (EZH2), and Naked Cuticle Homolog 2 (NKD2)	EMT signaling pathway	Cell proliferation, migration, metastasis, invasion, apoptosis suppression, and cell cycle control	Promoting	[20]
Gastric cancer	Paired tissues of 66 patients with GC and plasmas derived from 77 GC patients	AGS, SGC7901 and BGC-823 GC cell lines and a normal gastric cell line GES‐1	Upregulated	Epithelial markers CDH1, EpCAM, and mesenchymal markers CDH2, Vimentin, ZEB1, Snail, MMP14, and Twist	EMT	Regulating EMT and circulating tumor cells (CTC), TNM stage, cancer invasion, lymph node metastasis, and distant metastasis	Promoting	[21]
Gastric cancer	54 paired gastric cancers and normal tissues	BGC823 and SGC7901 cells	Upregulated	EZH2, LSD1/CREST, KLF2, and NKD2	–	Cell proliferation and apoptosis	Promoting	[22]
Glioma			Upregulated	MMP2, MMP9, N-cadherin, Integrin β1, ZEB1, Twist, and Snail down-regulation, and E-cadherin, Hes-1, and NICD over-expression	NOTCH and EMT signalling pathways	Cell proliferation, migration, and invasion	Promoting	[23]
Glioma	46 glioma patient samples and 11 normal brain tissue samples obtained from patients with severe head trauma	The human glioma cell lines (U87 and U251)	Upregulated	EMT-related proteins: N-cadherin, Snail, and E-cadherin, Notch signal-related proteins: Hes-1, and NICD	EMT and Notch signaling pathway	Glioma cell proliferation, migration, and invasion	Promoting	[24]
Osteosarcoma	Osteosarcoma tissues and corresponding noncancerous tissues from 50 patients	KHOS, 143b, LM7, U2OS, and MG-63	Upregulated	BMI1 and ZEB2	–	Promoted cell proliferation, migration, and invasion	Promoting	[25]
Epithelial ovarian cancer (EOV)	66 EOC tissue specimens and 10 normal ovarian epithelial tissues	OVCAR3, Caov3, OVCA429, SKOV3, A2780, and COV644	Upregulated	SP1	–	Cell proliferation, migration, and chemo-resistance	Promoting	[26]
Nonsmall cell lung cancer (NSCLC)	NSCLC tissue from 173 patients		Upregulated	–	–	Potential prognostic marker	Promoting	[27]
NSCLC	22 pairs of NSCLC and matched tumor-adjacent tissues	Two NSCLC cell lines (A549 and HCC827) and human bronchial epithelial cells (16HBE)	Upregulated	Cdc42	ZFAS1/miR-590-3p axis	Cell proliferation	Promoting	[28]
Squamous cell carcinoma	Tissues from 136 ESCC patients	EC9706, Eca109, TE-13, TE-1, and TTN	Upregulated	STAT3, miR-124	EMT	ZFAS1 induced the proliferation, migration, and invasion of esophageal squamous cell carcinoma (ESCC)cells and represses apoptosis	Promoting	[29]
ESCC	398 lymph node-negative ESCC patients	–	Upregulated	P53 CDK1/cyclin B1		Migration, invasion, metastasis	Promoting	[30]
Nasopharyngeal carcinoma (NPC)	Tumor tissues and matched adjacent tissues from 53 NPC patients and 62 NPC samples were obtained from The Cancer Genome Atlas (TCGA)	HONE-1, CNE-1, CNE-2, HNE-1, C666-1, HK-1, S26, S18, SUNE-1, 6-10B NP69, and N2Tert	Upregulated	METTL3	PI3K/AKT pathway EMT	NPC cell proliferation, migration, tumor growth, and autophagy	Promoting	[31]
Nasopharyngeal carcinoma	29 paired cancer tissues and corresponding adjacent normal tissues	CNE1, CNE2, HNE1, and HONE1	Upregulated	–	PI3K/AKT pathway	Cell proliferation, migration, and invasion, as well as apoptosis suppression	Promoting	[32]
Head and neck squamous cell carcinoma (HNSCC)	44 normal and 540 HNSCC tissue samples, including: oral cavity (*n* = 314), pharynx (*n* = 90) and larynx (*n* = 116) (cBioportal and UALCAN databases)	FaDu SCC-25, SCC-040, and dysplastic DOK cell lines	Upregulated	*ZNFX1*, *EIF4E*	EMT and Wnt pathways	Regulates cellular phenotypes, EMT process, proliferation, migration, invasion, and apoptosis	Promoting	[33]
Breast cancer	–	MCF-10A, T47D, MCF-7, MDA-MB-435, and BT-549	Down-regulated	miR-589	PTEN/PI3K/AKT pathway	Inhibition of proliferation, invasion, and migration of breast cancer cells by targeting miR-589	Suppressing	[34]
Breast cancer	–	MDA-MB-231, MCF-7, T-47D, and SK-BR-3	Down-regulated	–	EMT pathway	ZFAS1 reduced breast cancer cell proliferation, migration, and invasion and promoted cell cycle arrest and apoptosis	Suppressing	[35]
Clear cell renal cell carcinoma (ccRCC)	60 ccRCC tissues			SKA1		Proliferation and metastasis	Promoting	[36]
Pancreatic carcinoma (PC)	43 paired PC and adjacent equivalent specimens, GEPIA2 and the Cancer Genome Atlas (TCGA) databases	human PC cell strains (BxPC-3, PANC-1, SW1990, and PaCa-2) versus HPNE	Upregulated	HMGCR, U2AF2	Lipometabolism	PC cell multiplication and invasiveness	Promoting	[37]
PC	71 paired and 28 single PAAD tissue samples	SW1990, PANC1 and BXPC3, HEK-293T, and HPDE6C7	Upregulated	miR-3924/ROCK2		ZFAS1 promotes cell metastasis	Promoting	[38]
Cervical cancer (CC)	CC tissues and adjacent normal tissues from 85 cervical cancer patients	The CC CaSki and the HeLa cell lines	Upregulated			Cell proliferation, invasion, and migration	Promoting	[39]
CC	CC and the adjacent normal tissues from 53 female CC patients	293 T, SiHa, and CaSki cells	Upregulated	–	the miR-190a-3p/KLF6 axis	Cell proliferation and the enhancement of cervical tumor growth	Promoting	[40]
Endometrial carcinoma (EC)	64 pairs of ECs and adjacent tissues	HEC-1B, RL95-2, Ishikawa, and hEEC	Upregulated	CDK4, Cyclin-D1, N-cadherin, and E-cadherin	EMT	Cell proliferation, G1/S transition, migration, and invasion	Promoting	[41]
EC	60 pairs of EC and adjacent tissues	HEC-1B, Ishikawa, KLE, and RL-952	Upregulated	–	miR-34b/VEGFA axis	ZFAS1 promotes cell proliferation and metastasis	Promoting	[42]
Thyroid cancer	GSE50901, GSE3363, GSE29265, and TCGA datasets, including 58 matched normal tissues and 510 thyroid cancer samples	–	Upregulated	–	–	ZFAS1 promoted cell proliferation and cell cycle	Promoting	[43]
Melanoma	45 melanoma tissues and adjacent normal skin tissues	CHL-1, UACC904, A375, and 1205Lu	Upregulated	VIM, MMP2, MMP3, SMAD2, SNAIL2, TMEF1, ILK, CDH2, E-cadherin, and vimentin N-cadherin, CDH1, MST1R, CAV2, and KRT19	EMT process	Migration and invasion	Promoting	[44]
Acute myeloid leukemia		HL‑60, KG‑1, ML‑1, and SKNO‑1	Upregulated			Promotion of proliferation and inhibition of apoptosis	Promoting	[45]
Natural killer/T-cell lymphoma	17 NKTCL samples and 3 normal NK-cells (resting NK, PBNK48h, and NKCOD12)	NK92 and KHYG1	Upregulated	MDM2, an E3 ubiquitin protein ligase	Nuclear factor-kappa B signaling, β-catenin independent WNT signaling, and P53 pathway	Stabilization of P53 and regulation of apoptosis and cell cycle	Promoting	[46]
Prostate cancer	30 cancer tissues and paired adjacent tissues obtained from PCa patients	PC3, DU145, 22RV1, and LNCAP	Upregulated	E-cadherin N-cadherin and Snail	EMT process	Proliferation, migration, invasion stimulation, and apoptosis inhibition	Promoting	[47]
Bladder cancer	20 normal bladder tissue samples and 102 bladder cancer samples	T24, RT4	Upregulated	ZEB1, ZEB2, Vimentin, KLF2, NKD2, and E-Cadherin		Muscular invasion, lymph node metastasi, and distal metastasis	Promoting	[48]
Lung adenocarcinoma	46 pairs of LAD and nearby non-cancerous tissues	BEAS-2B, A549	Upregulated	FRS2	lncRNA ZFAS1/miR-1271-5p axis	Cell proliferation, migration, and invasion	Promoting	[49]

## ZFAS1 in apoptosis, cell proliferation, and cell cycle

The current gap in cancer management may be filled by understanding the role of ZFAS1 in apoptosis, cell proliferation, and the cell cycle. There are conflicting findings on ZFAS1's effect on apoptosis and cell proliferation. ZFAS1 regulates apoptosis and cell proliferation via inhibition of the Wnt/β-catenin signaling pathway and induces EMT via the Wnt/β-catenin pathway [50]. Silenced lncRNA ZFAS1 or boosted miR-129 lowered the apoptotic rate and expression of the pro-apoptotic Bax gene, whereas enhanced anti-apoptotic Bcl-2, E2, and P4 production promoted proliferation of ovarian granulosa cells in Polycystic Ovarian Syndrome (PCOS) [9]. According to another study, ZFAS1 reduced apoptosis in PC12 cells via the Bcl-2/Bax/cleaved caspase-3 pathway and might act as a cruciform-formable inverted repeat identifier (CIRI)-inhibiting repair gene [2].

According to the Li et al. study, downregulating ZFAS1 drastically decreased proliferation, induced cycle arrest in the G0/G1 phase, and increased tumor cell death in osteosarcoma cells [51]. Using flow cytometry, Xu et al. discovered that knocking down the ZFAS1 gene decreased cell proliferation and accelerated apoptosis in nasopharyngeal cancer cells [2].

Nie et al. used an MTT assay to show that gastric cancer cells transfected with si-ZFAS1 (si-RNA against ZFAS1) had poor growth and colon formation ability but a higher apoptotic rate compared to control cells, whereas ZFAS1 overexpression enhanced cell proliferation and colon formation while inhibiting apoptosis. Also, in vivo, ZFAS1 knockdown suppressed the development of gastric cancer cells [22]. Nie et al. used an MTT assay to show that gastric cancer cells transfected with si-ZFAS1 (si-RNA against ZFAS1) had poor growth and colon formation ability but a higher apoptotic rate compared to control cells, whereas ZFAS1 overexpression enhanced cell proliferation and colon formation while inhibiting apoptosis. Also, in vivo, ZFAS1 knockdown suppressed the development of gastric cancer cells [52].

Another study found that lowering the expression of ZFAS1 in triple-negative breast cancer (TNBC) cells increased the expression of mesenchymal cell markers like Slug and ZEB1 while lowering the expression of epithelial cell markers like E-cadherin, Claudin-1, and Zo-1, as well as affecting cell migration and invasion. ZFAS1 also boosted human TNBC cell proliferation and colonization by reducing the expression levels of the cyclin-dependent kinase (CDK) inhibitors p21 (CDKN1A) and p27 (CDKN1B). On the other hand, ZFAS1 and STAT3 were found to have a substantial negative association. In light of these findings, it is hypothesized that ZFAS1 influences TNBC progression by interfering with the STAT3 protein [53]. Fang et al. also discovered that ZFAS1 is downregulated in breast cancer tissues and cell lines, and overexpressed ZFAS1 can decrease cell proliferation and cause death by preventing the EMT process, so ZFAS1 may play a role in gynecological oncology [39].

ZFAS1 overexpression enhanced vascular smooth muscle cells (VSMCs) proliferation, migration, and invasion in response to oxidized low-density lipoprotein (ox LDL), as well as raised the expression of proteins involved in cell proliferation, migration, and invasion (Ki67, PCNA, MMP2, and MMP9). ZFAS1 knockdown partially reversed the effect of ox LDL treatment on VSMC proliferation, migration, and invasion, as well as the protein expression involved in cell proliferation, migration, and invasion [54].

The study by Guo et al. showed that mRNA ZFAS1 expression was increased in all four humans’ acute myeloid leukemia (AML) cell lines (HL-60, KG-1, ML-1, and SKNO-1) compared with the control cell lines (T lymphocytic leukemia or Burkitt's lymphoma). siRNA transfection into human AML cells down-regulated ZFAS1. The ZFAS1 effect on AML cell proliferation was evaluated by the A cell-counting kit-8 (CCK8) assay, and its effect on the cell cycle and apoptosis was assessed by flow cytometry. Based on the CCK-8 results, ZFAS1 knockdown blocked cell proliferation in HL-60 and SKNO-1 cell lines, and flow cytometry assays showed its knockdown led to AML cell cycle G1 phase arrest and apoptosis. So, ZFAS1 induced proliferation and prohibited the AML cells’ apoptosis [45].

ZFAS1 and apurinic/apyrimidinic exonuclease 1 (APEX1) were overexpressed, and miR-135a was down-regulated in osteosarcoma (OS) tissues and cells. ZFAS1 competitively bonded with miR-135a, which increased APEX1 expression. Moreover, blocked ZFAS1 or increased miR-135a prohibited colony proliferation formation, invasion, and migration but stimulated MG63 cell apoptosis and, finally, decreased OS tumor volume and weight in vivo [55].

In a contrast study, Askarian-Amiri et al. reported that, as ZFAS1 is unexpressed in invasive ductal breast carcinoma tissue, in comparison with normal breast tissue, ZFAS1 works as a tumor suppressor [56]. Fan et al. also determined the ZFAS1 underexpression with reverse transcription-quantitative polymerase chain reaction (RT-qPCR) and gain-of-function tests on breast cancer cell lines compared with controls [35]. Based on our literature review, generally, ZFAS1 acts as an oncogene, and only in two studies on breast cancer was it introduced as a tumor suppressor lncRNA.

## ZFAS1 in angiogenesis, invasion, and metastasis

The lack of knowledge about controlling cancer’s angiogenesis and invasion may be filled by understanding the ZFAS1 mode of action. Several researchers have uncovered the mechanisms through which ZFAS1 regulates cancer development. For example, CeRNA ZFAS1 can control cancer cells by sucking up miR-329, miR-150, and miR-48 [43]. When a tumor spreads to other parts of the body, many factors and biochemical processes are needed. Tumor cells become more aggressive and capable of distant metastasis when they undergo EMT, which is thought to be a precondition for this process. According to a study by Sharma et al., ZFAS1 silencing increased E-cad levels while decreasing N-cad and Snail levels. The loss or reduction of E-cad can alter EMT as an epithelial cell marker. Snail can decrease E-cad expression. Snail and N-cad overexpression are frequently associated with EMT. As a result, high-expressed ZFAS1 is involved in EMT and plays a vital role in cancer metastasis [53].

Furthermore, ZFAS1 expression was considerably elevated in colorectal cancer (CRC) tissues and cell lines and was related to Helicobacter pylori infection, lymph node metastases, advanced TNM stage, and poor overall survival in the suffered patients. In vitro and in vivo studies have shown that ZFAS1 inhibition can significantly reduce CRC cell growth and invasion. As a result, ZFAS1 promotes CRC metastasis by sponging miR-484 [57]. Also, ZFAS1 overexpresses in the advanced stages of CRC, leading to chemoresistance and changes in p53 expression. ZFAS1 blockage decreased DLD-1 and HCT-116 cell invasion and migration via the EMT process [58].

ZFAS1 induced colon cancer progression by competitively binding to the tumor suppressor miR-150-5p, which led to VEGF-A overexpression. ZFAS1 increased colon cancer cell proliferation, metastasis, angiogenesis, and EMT through the induction of the VEGFA/VEGFR2/Akt/mTOR signal transduction pathway [59]. Moreover, ZFAS1 expression levels were higher in patients suffering from cervix cancer with advanced FIGO stage, high histological grade, lymph node metastases, and deep myometrial invasion [39].

In a study, RT-PCR and lncRNA microarray tests showed that ZFAS1 was vigorously overexpressed in 3 pairs of osteosarcoma and their adjacent normal tissue. Moreover, ZFAS1 overexpression in 53 pairs of osteosarcoma patients was closely associated with a poor prognosis. In vitro analysis of ZFAS1 knockdown significantly blocked cell proliferation, arrested the cell cycle at the G0/G1 phase, and induced apoptosis. ZFAS1 knockdown can also inhibit cancer growth in vivo. Based on bioinformatics analysis, a luciferase reporter assay, and an RNA immunoprecipitation (RIP) assay, ZFAS1 can sponge miR-486 at its 3,-UTR as a ceRNA. Furthermore, based on rescue analysis, miR-486 could reverse the ZFAS1 effect on osteosarcoma genesis [51].

In addition, according to RNA sequencing data, ZFAS1 expression was upregulated in gastric cancer tumor tissues relative to normal tissues [22]. The migration and invasion of stomach cancer cells were detected using the Transwell test in another study. The results indicated that knocking out ZFAS1 reduced migration and invasion rates by 40% and 44%, respectively, and the expression of MMP-2 and MMP-14, which play essential roles in cell invasion, was reduced. In ZFAS1-silenced gastric cancer cells by 44% and 74%, respectively. Thus, ZFAS1 knockdown inhibited migration, invasion, and EMT in gastric cancer cells [52].

## The role of ZFAS1 in signaling pathways

The mechanisms underlying the effects of ZFAS1 are complex and involve multiple signaling pathways. Recent research has discovered ZFAS1 amplification in hepatocellular carcinoma (HCC) and CRC. ZFAS1 binds to CDK1 to control the p53-dependent cell cycle and apoptosis in CRC cells, as well as promotes HCC cell metastasis by binding to miR-150 and reversing its tumor-suppressive effect [22].

In a study of gastric cancer tissues, disruption of Wnt signaling has been related to gastric carcinogenesis and nuclear β-catenin accumulation in 20–30% of gastric cancer tumors. Studies demonstrated that following ZFAS1 silencing, the cellular and nuclear protein levels of β-catenin and GSK3 phosphorylation (Ser9) decreased, while the cellular NKD2 level enhanced, and Wnt signaling was deactivated. Wnt signaling is critical for cell proliferation, differentiation, and migration. WNT gene products can bind to different receptors and trigger a variety of downstream signaling pathways, including the canonical Wnt/β-catenin pathway [52].

ZFAS1 presents in both the cytoplasm and the nucleus, with a higher ratio of nuclear ZFAS1, and in gastric cancer cells, direct binding of ZFAS1 to enhancer of zeste homolog 2 (EZH2), lysine-specific demethylase 1 (LSD1), and REST corepressor 1 (CoREST) was confirmed via RIP tests. Furthermore, EZH2 or LSD1 down-regulation increased KLF Transcription Factor 2 (KLF2) and NKD Inhibitor of WNT Signaling Pathway 2 (NKD2), whereas ZFAS1 under-expression caused EZH2 or LSD1 binding to the KLF2 or NKD2 gene promoter. Therefore, ZFAS1 works as an oncogene in gastric cancer by blocking KLF2 and NKD2.ZFAS1 presents in both the cytoplasm and the nucleus, with a higher ratio of nuclear ZFAS1, and in gastric cancer cells, direct binding of ZFAS1 to enhancer of zeste homolog 2 (EZH2), lysine-specific demethylase 1 (LSD1), and REST corepressor 1 (CoREST) was confirmed via RIP tests. Furthermore, EZH2 or LSD1 down-regulation increased KLF Transcription Factor 2 (KLF2) and NKD Inhibitor of WNT Signaling Pathway 2 (NKD2), whereas ZFAS1 under-expression caused EZH2 or LSD1 binding to the KLF2 or NKD2 gene promoter. Therefore, ZFAS1 works as an oncogene in gastric cancer by blocking KLF2 and NKD2 [22].

Moreover, ZFAS1 activates the EMT pathway as a key pathway in the initiation and development of tumors via interaction with ZEB2 to stabilize it. This pathway contains several markers such as MMP2, MMP9, E-cadherin, N-cadherin, Integrin β1, ZEB1/2, Twist, and Snail, which were vigorously underexpressed along with ZFAS1 silencing [60].

ZFAS1 silencing in glioma cells led to the down-regulation of Hes-1 and NICD as important regulators of the Notch signaling pathway. So, ZFAS1 can control proliferation and apoptosis via this axis [61–63]. ZFAS1 also activates the oncogene p53 pathway in different cancers. The p53 protein acts as a nuclear transcription factor, regulating DNA repair, cell proliferation, and apoptosis. Decreased ZFAS1 is attributed to cell cycle arrest and apoptosis induction via the under-expression of cyclin B1 and p53 as well as the induction of PARP cleavage [64].

Furthermore, ZFAS1 was significantly overexpressed in nasopharyngeal carcinoma (NPC) tissues and cell lines. ZFAS1 knockdown significantly blocked cell proliferation and invasion, arrested cell cycle progression, and induced cell apoptosis, as well as decreased EMT. 740Y-P could also reverse the effects of ZFAS1 downregulation on apoptosis, proliferation, and invasion in 5-8F cells. So, ZFAS1 could act as an oncogene in NPC and promote cell proliferation and invasion through the PI3K/AKT pathway in NPC cells [32].

ZFAS1 was also overexpressed in OC and induced cell proliferation, invasion, migration, and reduced cisplatin sensitivity by direct miR-548e sponging. ZFAS1 is localized with miR-548e in the cytoplasm of these cells. miR-548e booked CXCR4 and let-7a/BCL-XL/S Signaling Axis [65].

ZFAS1 was increased in HCC, causing malignancy and a worse prognosis with shorter survival in patients suffering from HCC. By silencing ZFAS1, the HCC malignancy of cells was prohibited, whereas miR-624 inhibitors could somehow restore the repressive effect of si-ZFAS1. On the other hand, ZFAS1 bonded to midkine (MDK) via miR-624, which stimulated the extracellular-regulated protein kinases/c-Jun N-terminal kinase (ERK/JNK)/P38 signaling pathway; therefore, HCC was promoted [66].

## The role of ZFAS1 in chemoresistance

Effective chemotherapies will increase oral survival and the quality of life of numerous cancer patients worldwide. Chemotherapies cause tumor cells’ death by generating DNA damage, preventing its repair, stopping the cell cycle, and increasing apoptosis effectively. After surgical resection, chemotherapy decreases the remaining tumor and avoids recurrence, with a higher 5-year survival rate in recent decades [67]. Despite the efficacy of chemotherapy, inherent and acquired chemoresistance provide a significant obstacle to cancer treatments [8]. Resistance to chemotherapeutics is still a significant issue in cancer treatment [67].

Different agents can affect cancer cell responses to various treatments. For example, ZFAS1 has the potential to improve the response of cancer cells to conventional treatment approaches by altering the responsiveness of cancer cells to radiotherapy and chemotherapy, as shown in several studies. Furthermore, si-ZFAS1 might significantly improve CaSki and HeLa cell chemosensitivity to cisplatin [39]. ZFAS1 could also increase glioma cells' cisplatin cytotoxicity and vitality via direct miR-432-5p sponging [68].

ZFAS1 was one of eight lncRNAs that was significantly associated with chemosensitivity among the lncRNA profiles of 258 high-grade serous ovarian cancer (HGS-OvCa) patients (AUC = 0.83). The relationship of the mentioned marker with differentiated, mesenchymal, and immunoreactive subtypes revealed its excellent prognostic potential (AUC > 0.8). A significant association between the ZFAS1 pattern and chemosensitivity was indicated in 233 HGS-OvCa patients. In addition, cisplatin upregulated ZFAS1 in HeyA8, HeyC2, and A2008 cell lines. So, ZFAS1 may lead to platinum resistance [69]. LncRNA ZFAS1 is also associated with GC progression and resistance to chemotherapeutics such as paclitaxel (PTX) and cisplatin. So, ZFAS1 can be a diagnostic candidate or therapeutic target of GC [52].

Wang et al. suggested a novel mechanism of cisplatin resistance via the ZFAS1/miR-421/MEIS2 axis on OSCC cells and a nude mouse xenograft model. As ZFAS1 overexpression significantly improved OSCC cell proliferation and increased cell survival in cisplatin-resistant cells, it may regulate caspase-3 activities as well as BAX and BCL2 expression in cisplatin-resistant OSCC cells. By sponging miR-421 and modulating MEIS2 expression, ZFAS1 improved the chemoresistance of OSCC to cisplatin [70].

In a study, RSV could adjust PTX resistance and mitophagy in NSCLC through the ZFAS1/miR-150-5p-mediated PINK/Parkin pathway. Based on Luciferase activity, ZFAS1 has a direct interaction with miR-150-5p to control the expression of PTEN-induced putative kinase 1 (PINK1) as an important mitophagy regulator in NSCLC. Furthermore, ZFAS1 was a downstream effector of resveratrol (RSV), which is a natural regulator of mitochondrial metabolism. So, based on the ZFAS1 role, simultaneous administration of these two drugs may be a novel NSCLC treatment [71].

In pediatric acute myeloid leukemia (AML), ZFAS1 elevates resistance to Adriamycin (ADR) as a frequent chemotherapeutic. According to studies, ADR promotes ZFAS1 in pediatric AML, and knockdown of ZFAS1 or Myb diminishes ADR resistance in vitro [72]. ZFAS1 directly interacts with miR-195, which regulates Myb to increase ADR resistance in pediatric AML [73]. Therefore, ZFAS1 may be a diagnostic or therapeutic marker for ADR resistance in pediatric AML [72].

In another study, ZFAS1 directly controlled miR-150-5p, which in turn increased ovarian cancer growth via modulating Sp1, and findings revealed that ZFAS1 was necessary for EOC cell chemoresistance, but miR-150-5p rendered EOC cells more vulnerable to Cisplatin and Paclitaxel [26]. ZFAS1 may also play a role in other chemoresistance in EOC cells [74]. Moreover, in lung adenocarcinoma, ZFAS1 induces proliferation, migration, and chemoresistance [74]. So, ZFAS1 can act as a diagnostic or therapeutic marker for cisplatin or other drug resistance in these cancers. Table 2 shows the ZFAS1-related chemoresistance in several cancers.

**Table 2:** *ZFAS1* associated chemoresistance in different cancers

Chemotherapeutic resistance	Cancer type	References
Cisplatin resistance	Ovarian cancer	[75]
	Cervical cancer	[39]
cis-platinum or Paclitaxel resistance	Gastric cancer	[52]
Adriamycin (ADR) resistance	T-cell acute lymphoblastic leukemia	[58]

## The significance of ZFAS1 in cancer diagnosis and prognosis

A biomarker is a characteristic that can be objectively assessed as an indicator of normal or pathological [76]. Biomarkers can be divided into diagnostic, prognostic, predictive, and therapeutic. Diagnostic biomarkers can detect a disorder in a noninvasive situation as they are usually assessable in high amounts in clinical samples of patients [77]. Prognostic biomarkers predict the probability of recurrence or progression of diseases, and can help in making decisions [78] and therapeutic biomarkers can be targeted in a therapeutic process [77].

Accurate cancer diagnostic and prognostic markers can be found by studying the ZFAS1 mode of action. In cancer cells, ZFAS1 knockdown drastically slowed the cell cycle by increasing the number of G1 phase cells and lowering the number of S and G2/M phase cells. Bioinformatics analysis showed that translation, rRNA processing, and spliceosomal snRNP assembly regulation are linked to ZFAS1 positively co-expressing genes. Additionally, protein deubiquitination, forebrain development, peptidyl-serine phosphorylation, and vesicle-mediated transport were associated with ZFAS1 negatively co-expressing genes [43].

Regarding these regulatory roles of ZFAS1 in various cancers, it has the potential to be a diagnostic or prognostic marker (Table 3). For instance, increased ZFAS1 expression was linked to gastric tumor size, advanced pathological stage, and poor prognosis. Patients with higher ZFAS1 levels experienced a shorter overall survival than those with lower ones [22]. ZFAS1 expression was also significantly higher in HCC patients and was linked to intrahepatic and extrahepatic metastases, as well as a poor prognosis. ZFAS1 was introduced as a new diagnosis marker for HCC. In a study on HCC, ZFAS1 was analyzed in the plasma of 79 healthy controls and 60 HCC patients. The HCC plasma had significantly higher ZFAS1 levels than healthy controls (P < 0.001), and the ZFAS1 AUC was 0.801 for the HCC diagnosis compared to healthy controls [79].

**Table 3:** Diagnostic/prognostic role of *ZFAS1* in various types of cancers

Sample number	Area under curve	Sensitivity	Specificity	Kaplan–Meier analysis	Univariate cox regression	Multivariate cox regression		References
Plasma-derived from 77 gastric cancer patients	0.727	0.766	0.766	**–**	**–**	**–**		[21]
54 paired gastric cancers and normal tissues	**–**	**–**	**–**	Patients with higher ZFAS1 levels had a shorter OS and PFS time than those with low ZFAS1 levels	Increased ZFAS1 expression was correlated with tumor size and advanced pathological stage in gastric cancer	**–**		[22]
70 pairs of matched hepatoblastoma and normal tissues	**–**	**–**	**–**	Overexpression of ZFAS1 correlates with clinicopathological features and a poor prognosis in HB patients	High ZFAS1 expression was a potent independent risk indicator for survival in HB patients			[83]
88 cases of patients with hepatocellular carcinoma	**–**	**–**	**–**	Upregulation of ZFAS1 is correlated with a poor prognosis in HCC	Upregulation of ZFAS1 is more frequently observed in HCC patients with microvascular invasion than in those with no microvascular invasion, though there is no significant correlation between ZFAS1 expression and gender, age, tumor number, tumor size, AFP level, or HCC stage			[18]
Cervical cancer tissues and adjacent normal tissues from 85 cervical cancer patients	**–**	**–**	**–**	Overexpression of ZFAS1 correlates with a poor prognosis in patients with cervical cancer				[39]
Cervical cancer tissues and the adjacent normal tissues from 53 female CC patients	**–**	**–**	**–**	Higher ZFAS1 expression group had a lower 5-year survival rate	FIGO staging and tumor diameter was correlated with ZFAS1 expression			[40]
46 glioma patient samples and 11 normal brain tissue samples obtained from patients with severe head trauma	**–**	**–**	**–**	Glioma patients with high ZFAS1 expression had a poor OSl compared with patients with low ZFAS1 expression	ZFAS1 expression was increased in patients with a high WHO grade (III–IV) compared to patients with a low WHO grade (I–II)			[24]
44 normal and 540 Head and Neck Squamous Cell Carcinoma tissue samples	**–**	**–**	**–**	Patients with a lower expression of *ZFAS1* presented a slightly longer PFS and OS	Gender, T, N, invasion, and HPV p16 status-caused differences in survival time were statistically significant			[33]
Osteosarcoma tissues and corresponding noncancerous tissues from 50 patients	**–**	**–**	**–**	ZFAS1 reduced the survival rate of osteosarcoma patients				[25]
398 lymph node-negative ESCC patients		~ 0.2	~ 0.7	ESCC patients with high ZFAS1 expression had a poor OS	Histological grade, T stage, and ZFAS1 expression significantly affected OS	Histological grade, T stage, and ZFAS1 expression were independent risk factors for OS		[30]
66 EOC tissue specimens and 10 normal ovarian epithelial tissues	**–**	**–**	**–**	The EOC patients with high ZFAS1 had a poor prognosis				[26]
53 nasopharyngeal carcinoma patients	**–**	**–**	**–**	patients with high ZFAS1 expression had worse OS and PFS				[31]

(OS: overall survival, PFS: progression-free survival)

Moreover, in colorectal cancer, upregulated ZFAS1 can operate as an oncogene by destabilizing p53 or interacting with CDKl/cyclin B1 to increase the cell cycle and suppress cell death [80]. ZFAS1 expression was elevated in cervical cancer tissues, and patients with low ZFAS1 expression had a considerably greater survival rate than those with high one [39].

In a study, the expression of ZFAS1, miR-497-5p, and HMGA2 in pancreatic cancer (PaC) tissues was assessed by qRT-PCR, and ZFAS1 biological roles were investigated by CCK8, EdU, transwell, and scratch wound assays. ZFAS1 mechanisms were found by MS2-RIP, RNA pull-down, RNA-ChIP, and luciferase reporter assays. Based on these results, ZFAS1 was unregulated in xenograft PaC tissues, leading to PaC growth, and targeted HMGA2 via the decoying tumor suppressor miR-497-5p. So, as ZFAS1 induces PaC progression in vivo via adjusting the miR-497-5p/HMGA2 axis, it acts as a PaC therapeutic target and diagnostic marker [81].

Also in another study, ZFAS1 was unregulated in the pulmonary tissues of rats suffering from bleomycin (BLM)-induced pulmonary fibrosis (PF). ZFAS1 blocking prohibited BLM-induced PF by preventing FMT and lipid peroxidation. ZFAS1 blocked SLC38A1 as a ceRNA via sponging miR-150-5p [82]. Moreover, bZIP-family tumor-inducing factor (TF) CREB3 can induce ZFAS1 expression via direct binding to its promoter. The CREB3 family, like CREB3, affects cell metabolism, division, and cancer via binding to downstream factors like CCR1 and HDAC3. CREB3-promoted ZFAS1 controls the miR-373-3p/MMP3 axis via thyroid cancer metastasis [3]. As ZFAS1 was elevated in the early stages of thyroid cancer, it can be considered a thyroid cancer prognostic factor [43].

## Crosstalk between ZFAS1 and miRNAs

lncRNAs can control gene expression by interacting with RNA-binding proteins or acting as endogenous competitors for miRNAs. ZFAS1 also regulates human cancer through interaction with miRNAs. For example, ZFAS1 binds directly to miR-1271-5p as a molecular sponge in lung adenocarcinoma (LAD) cells. Therefore, ZFAS1 overexpression blocks the inhibitory function of miR-1271-5p in LAD cells, such as blocking cell proliferation, invasion, and migration [49].

A conflicting investigation showed ZFAS1 under-expression and miR-589 over-expression in breast cancer cells. By ZFAS1 upregulation, the PTEN/PI3K/AKT pathway prohibits cell proliferation, invasion, and migration, whereas by miR-589 upregulation, these functions decrease. In breast cancer, ZFAS1 can lead to increased apoptosis through PTEN activation as a PI3K/AKT inhibitor, while miR-589 reverses ZFAS1 functions [34].

Tumor suppressor miR-34b directly inhibited ZFAS1 via targeting ZFAS1 3′UTR and oncogene SOX4. SOX4 was positively associated with ZFAS1. On the other hand, ZFAS1 silencing overexpressed SOX4 in DLD-1 cells via targeting miR-34b [58].

Upregulated ZFAS1 was inversely attributed to miR-7-5p expression and was responsible for better overall survival in CRC tissues. When ZFAS1 targeted miR-7-5p in CRC, tumor development, invasion, and migration were blocked, whereas apoptosis was induced [84].

Bioinformatics analysis showed that the seed sequences of miR-582-3p are the potential targets of ZFAS1 3′-UTR. miR-582-3p Upregulation reduced ZFAS1's luciferase function. ZFAS1 knockdown increases miR-582-3p-induced inflammation, apoptosis, and oxidative stress. So, lncRNA ZFAS1 was considered a negative regulator of miR-582-3p [2]. Table 4 presents some of the interactions between ZFAS1 and miRNAs in various cancers.

**Table 4:** Interactions between *ZFAS1* and miRNAs in different cancers

miRNAs	Cancer type	Function	References
miR-484	Colorectal cancer	Proliferation inhibition, apoptosis induction, suppression of migration, invasion, and the EMT process through the down-regulation of ZEB1 and SMAD2	[17]
miR-590-3p	Colorectal cancer	Cell proliferation (sponging leads to G1-arrest)	[15]
	Non-small cell lung cancer	ZFAS1 inhibits cell division cycle 42 (Cdc42) expression by regulating miR-590-3p	[28]
miR7-5p	Colorectal cancer	Proliferation, migration, invasion, and apoptosis	[84]
miR-150	Hepatocellular carcinoma	Suppression of HCC cell invasion and metastasis by targeting ZEB1, MMP14, and MMP16	[18]
	T-cell acute lymphoblastic leukemia	Sialylation modulation of EGFR	[58]
	Ovarian cancer	Suppression of transcription factor SP1 expression	[26]
	Colorectal cancer	Suppression of cell proliferation, migration, invasion, and angiogenesis. Competitive binding of ZFAS1 to miR150-5p reduces the latter’s tumor suppression effect by targeting VEGFA	[85]
	Head and neck squamous cancer	Upregulation of EIF4E	[33]
	Melanoma	Promotion of tumorigenesis through regulation of the miR-150-5p/RAB9A axis	[44]
miR200b miR200c	Osteosarcoma	Competitive binding of ZFAS1 to miR-200b/c increases B lymphoma Mo-MLV Insertion Region 1 Homolog (BMI1) expression	[25] [86]
miR-548e	Ovarian cancer	Regulation of metastasis and cisplatin resistance	[65]
miR-589	Breast and gastric cancer	Targeting PTEN and reducing the effect of ZFAS1 on the inhibition of breast cancer cell proliferation, migration, and invasion	[34]
miR-10a	Clear cell renal cell carcinoma	SKA1 downregulation	[36]
miR-124	Esophageal squamous cell carcinoma	STAT3 upregulation	[87]
miR-940	Prostate cancer	miR-940 indirectly targets GAS5 and ZFAS1 via NAA10 and RPL28	[88]
miR-193a-3p	Hepatoblastoma	HB cell mobility: ZFAS1 regulates RALY expression by sponging miR-193a-3p	[19]
	Cervical cancer	Overexpression of ZFAS1 and inhibition of miR-190a-3p increase the expression levels of KLF6 and tumor progression	[40]
	Hepatocellular carcinoma	ZFAS1 enhances the proliferation of HCC cells by suppressing miR-193a-3p	[89]
miR-135a	Prostate cancer	It inhibits cell proliferation, invasion, and the EMT process and promotes apoptosis	[47]
	Nasopharyngeal cancer		[90]
miR-3924	Pancreatic Adenocarcinoma	Inhibition of metastasis	[91] [11]
miR-1271-5p	Lung adenocarcinoma	Tumor suppression by targeting FRS2	[49]
miR-34b	Endometrial carcinoma	Regulates the expression of vascular endothelial growth factor A (VEGFA)	[42]
miR-100-3p	Nasopharyngeal carcinoma	miR-100-3p can inhibit ATG10 expression (involved in autophagy), and this regulatory effect can be reversed by ZFAS1	[31]

## Conclusion and future perspectives

This comprehensive review has evaluated the role of ZFAS1 in various cancers’ chemoresistance, cell cycle, apoptosis, proliferation, invasion, and metastasis to find its potential as a diagnostic, prognostic, or therapeutic agent.

The ZFAS1 crosstalk with cellular signaling pathways such as Wnt¸ EMT, PTEN/PI3K/AKT, Notch, and p53 pathways confirms its key role in tumorigenesis. Based on multiple reports, in most cases, ZFAS1 was amplified or upregulated in human malignancies, accompanied by worse prognosis, like overall survival and metastasis to the lymph node. In vitro ZFAS1 knockdown inhibited cell proliferation and invasion, as well as induced apoptosis.

Moreover, in vivo experiments confirmed that ZFAS1 silencing would repel tumorigenesis. ZFAS1 could be considered a ceRNA at the molecular level because of its gene regulation function via competing with related microRNAs.

A few causative mechanisms of ZFAS1 in various cancers have been recognized up to now, but other ones should be discovered too. And because of the presence of some of the lncRNAs in body fluids, like urine and plasma, as well as their potential as noninvasive cancer-specific biomarkers in early tumor diagnosis, prognosis, and treatment.

Even though the use of ncRNAs in diagnosis and treatment suffers from some limitations, for example, target specificity should be ensured to evade off-target effects [92]. The next problem is ncRNA delivery. Local delivery of ncRNAs at the subcellular level poses a significant challenge for their therapeutic application. The stability of ncRNA is also another issue that impacts its functionality [93]. To overcome these problems, delivery tools can be improved. For example, nanoparticles increase the specific delivery of ncRNAs [94]. Furthermore, more efficient stabilizing effectors and chemistries increase ncRNA stability [94]. Technical improvement can also elevate tissue-specific targeting of ncRNAs while decreasing off-target ones [94]. Moreover, fine-tuning treatment amounts with specific inhibitors or activators can adjust ncRNA quantity effectively for therapy [93]. These limitations and strategies emphasize the complexity and therapeutic potential of ncRNAs as diagnostic or therapeutic markers, highlighting the need for novel research to manage their application. However, deeper investigations will discover the molecular mechanism of ZFAS1 in each cancer and also determine its clinical usage.

## Data Availability

No data was used for the research described in the article.
